# An Invasive Solitary Fibrous Tumor/Hemangiopericytoma Originating From the Falx: A Case Report

**DOI:** 10.7759/cureus.75850

**Published:** 2024-12-17

**Authors:** Ozan Başkurt, Selim Şeker, Idris Avci

**Affiliations:** 1 Neurosurgery, İstinye University Faculty of Medicine, Istanbul, TUR; 2 Neurosurgery, İstinye University Liv Hospital Bahcesehir, Istanbul, TUR; 3 Neurosurgery, Npistanbul Brain Hospital, Istanbul, TUR

**Keywords:** brain tumor, dural sinus, hemangioperistoma, intracranial solitary fibrous tumor, microsurgical resection

## Abstract

Intracranial solitary fibrous tumors (SFTs) and hemangiopericytomas (HPCs) are rare, aggressive tumors typically found along the dural sinuses. Despite their aggressive nature, complete surgical resection remains the most significant factor in reducing recurrence and improving survival. Here, we present the case of a 32-year-old male patient who presented with a new-onset headache and vertigo. Magnetic resonance imaging revealed a 50×37 mm left occipital mass originating from the falx and extending into the tentorium, with heterogeneous enhancement and compression/invasion of the left transverse sinus. Total resection was accomplished using microsurgical techniques, and histopathological analysis confirmed SFT/HPC, classified as WHO Grade II. Given the complete resection, including the venous sinus involvement, adjuvant radiotherapy was deemed unnecessary by the multidisciplinary committee. At the latest follow-up, five years post surgery, the patient showed no residual tumor or recurrence, with a reported overall well-being. Early detection and aggressive management through total resection can significantly improve survival and prognosis in patients with SFTs/HPCs.

## Introduction

Solitary fibrous tumors (SFTs) were first described in 1931 by Klemperer and Rabin as primary spindle cell tumors of the pleura [1]. While they predominantly affect the visceral pleura, their occurrence in the central nervous system (CNS) is rare, first documented by Begg and Garret in 1954 [2]. Intracranial SFTs and hemangiopericytomas (HPCs), account for less than 1% of all intracranial tumors and are thought to arise from the meninges [3-6].

Initially considered histologically benign, research has demonstrated their potential for aggressive behavior, including anaplastic transformation, multiple recurrences, and metastatic potential, classifying them as malignant neoplasms with sarcomatous characteristics [7,8].

Diagnosis is challenging due to their resemblance to other intracranial tumors, and histopathological confirmation remains the gold standard [9,10]. Currently, there is no consensus on the optimal therapeutic strategy [10]. This report presents a case of a falxo-tentorial SFT/HPC with sinus invasion and its management.

## Case presentation

**Figure 1:**
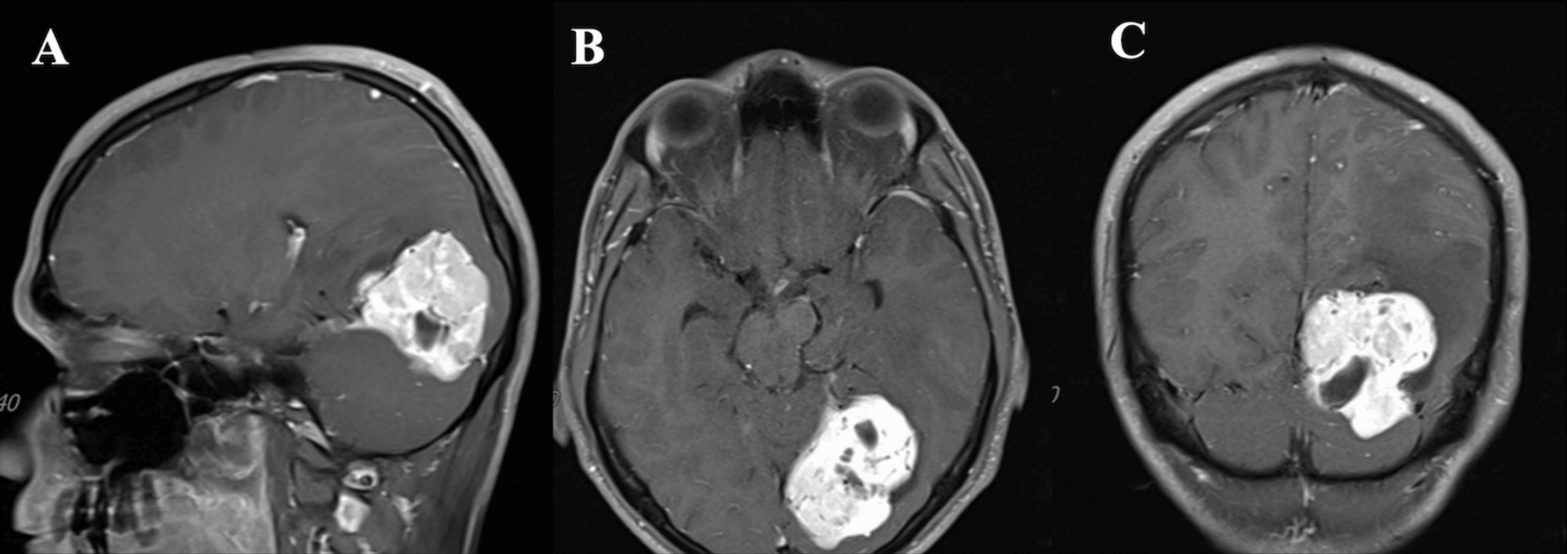
Preoperative brain MRI. Sagittal (A), axial (B), and coronal (C) gadolinium-enhanced T1WI showing a large tumor in left occipital region, originating from the falx, extending into the tentorium, and exhibiting heterogeneous enhancement.

A 32-year-old male patient presented with a new-onset headache and vertigo. Both physical and neurological examinations revealed no abnormalities. Magnetic resonance imaging (MRI) identified a 50×37 mm left occipital mass originating from the falx and extending into the tentorium. The mass exhibited heterogeneous enhancement and had a close relationship with the left transverse sinus (Figure 1).

**Figure 2:**
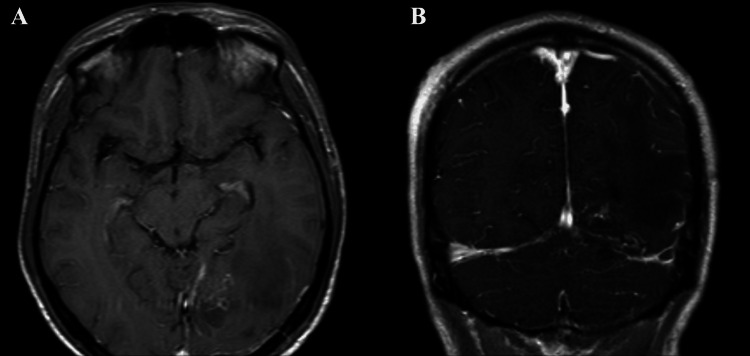
Postoperative brain MRI. Enhanced T1WI in axial (A) and coronal (B) showing the tumor was totally resected.

A left occipital craniotomy was performed using a posterior interhemispheric approach. The tumor was highly vascularized and well-demarcated, situated directly above the transverse sinus. Due to intraoperative findings of the tumor's invasion of the transverse sinus (on the non-dominant side), the sinus was excised and bleeding was controlled using silver clips. The texture of the tumor was characterized by good cleavage from the parenchyma, firm consistency, abundant neovascularization, and a grayish-red appearance. The descending portion of the tumor, extending into the tentorium, was resected with a cranial ultrasonic surgery aspirator. Complete resection was achieved using microsurgical techniques (Figure 2).

**Figure 3:**
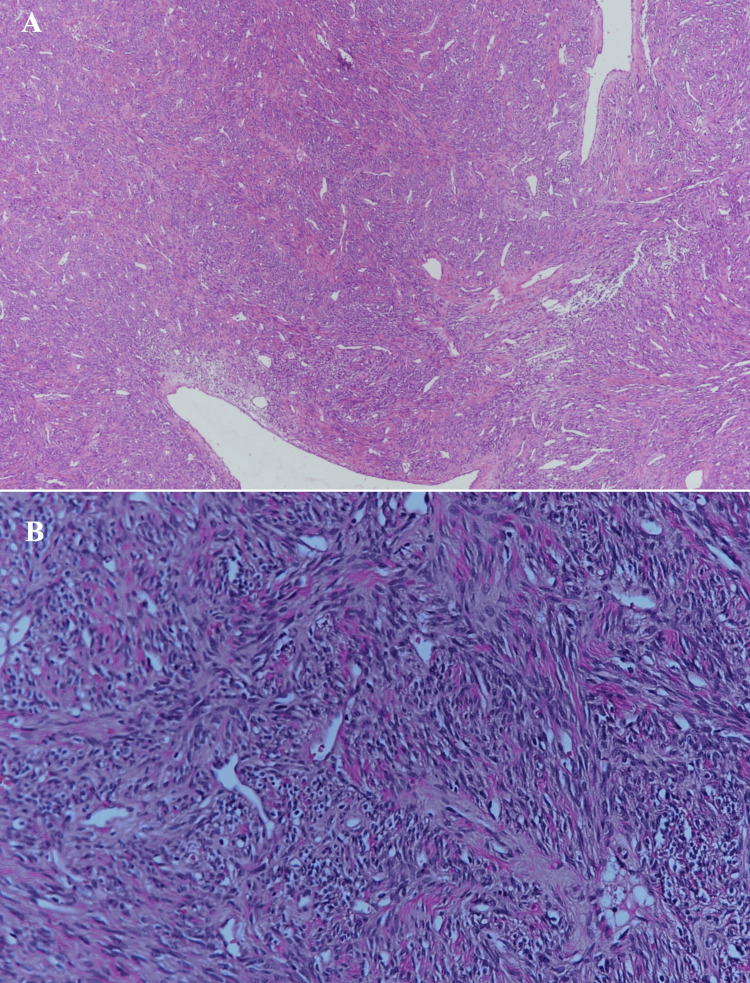
Spindle to oval cells, “staghorn” vascular pattern, biphasic architecture, and hyalinized vessels with hematoxylin and eosin staining (Magnification x100 (A) and x200 (B))

Histopathological examination revealed a malignant tumor with diffuse structure and high cellularity, composed of spindle to oval cells, a characteristic “staghorn” vascular pattern, biphasic architecture, and hyalinized vessels (Figure 3).

**Figure 4:**
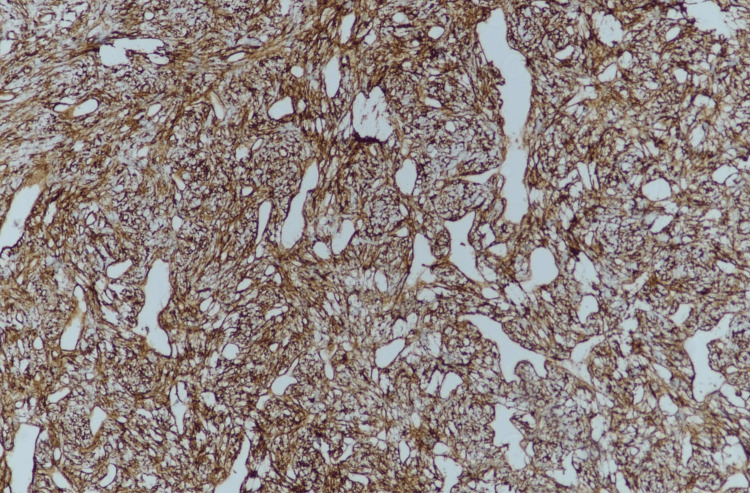
Immunohistochemistry exhibited positive immunostaining for CD34.

Additional immunohistochemical analysis showed four mitoses per 10 high-power field (HPF), CD34 positivity, epithelial membrane antigen (EMA) negativity, S-100 negativity, and a Ki-67 index of 3% (Figure 4).

The final diagnosis was intracranial SFT/HPC, classified as WHO 2016 Grade II [11].

Given the complete resection, including involvement of the adjoining venous sinus, and the WHO Grade II pathology, adjuvant radiotherapy was deemed unnecessary by the multidisciplinary tumor council. Postoperatively, the patient experienced only a mild headache during the first week, which resolved without intervention. No additional neurological deficits were observed. At the five-year follow-up, the patient remained tumor-free, with no recurrence, and reported overall well-being.

## Discussion

The primary epidemiological insight regarding CNS SFTs/HPCs is that they typically present during the fourth decade of life, with a higher prevalence in men [12,13]. However, Champeaux Depond et al. found that SFTs/HPCs are equally distributed between men and women, with most cases diagnosed around 50-55 years of age [14]. This multi-center epidemiological study included 399 histologically confirmed cases of newly diagnosed meningeal SFTS/HPCs in France over a 10-year period. Unfortunately, data regarding the specific anatomical locations were not provided. Redaelli et al. evaluated 82 patients pathologically diagnosed with SFT/HPC over a 39-year period [15]. Approximately 60-80% of these tumors occur in the supratentorial region [16,17], as seen in our case.

A key feature of SFTs/HPCs is their aggressive local growth, high recurrence rate, and their distinct potential for metastasis [12,18-20]. Histopathologically, these tumors were initially debated as either mesothelial or mesenchymal in origin [17]. However, recent immunohistochemical and electron microscopic studies strongly suggest that they originate from mesenchymal fibroblast-like cells [3,6,11,16,21].

The clinical presentation of SFTs/HPCs varies, with headache and focal neurological deficits such as paresis and seizures being common symptoms. In our case, the patient's presentation of headache and vertigo was attributed to an intracranial space-occupying lesion causing increased intracranial pressure and compression of the transverse sinus [16,22]. 

Radiologically, SFTs/HPCs can be challenging to differentiate from more common tumors such as meningiomas, schwannomas, or gliomas [17]. SFTs/HPCs tend to exhibit more heterogeneity than meningiomas [8]. However, higher-grade meningiomas also become increasingly heterogeneous. MRI of SFTs/HPCs typically reveals a lobulated and/or irregular cross-leaf-shaped mass without calcification or hyperostosis. Atypical SFTs/HPCs, on the other hand, may present with prominent brain edema, frequent bone destruction, and a narrow base of the “dural tail” features also seen in meningiomas [23]. Therefore, additional radiological evaluations, such as angiographic studies, may be employed to demonstrate small, irregular vessels with a "corkscrew" appearance, which is characteristic of SFTs/HPCs [4,16,20,24]. In our case, no further radiological evaluation was pursued, as the treatment strategy for both atypical high-grade meningiomas and SFTs/HPCs remains the same: radical surgical resection.

Despite their aggressive nature, complete surgical resection remains the most significant factor in reducing recurrence and improving survival [13,17]. Patients who undergo total or gross total resections have longer progression-free survival time compared to those with subtotal resections [16,21,25]. The rate of gross total resection depends on factors such as tumor size, involvement of major blood vessels, and intraoperative bleeding [10]. The use of silver neuro clips was instrumental in achieving total resection during the surgery of our patient.

Although adjuvant radiotherapy is not universally recommended, it has been shown to improve local control and reduce recurrence rates, particularly in cases of subtotal resection [8,21,26-29]. Schiariti et al. reported that adjuvant radiotherapy extended the local recurrence interval from 154 months to 254 months in patients following surgery [29]. In our patient, given the complete resection, including venous sinus involvement, and the WHO Grade II pathology, the multidisciplinary tumor council deemed adjuvant radiotherapy unnecessary.

## Conclusions

Intracranial SFTs/HPCs are rare and often pose a diagnostic challenge due to their resemblance to more common brain tumors. Histopathological analysis is essential for accurate diagnosis. Aggressive surgical resection is the cornerstone of treatment, as it significantly improves prognosis and reduces recurrence rates. While there is no consensus on the optimal therapeutic strategy, especially for tumors that are difficult to completely resect, surgical intervention remains crucial.

We recommend that surgery be considered as the primary treatment approach for these tumors, with careful planning to address potential complications, such as involvement with venous sinuses or surrounding structures. Future research should aim to establish a comprehensive surgical and therapeutic algorithm for managing these challenging tumors.
